# CircCOL1A1 promotes proliferation, migration, and invasion of colorectal cancer (CRC) cells and glutamine metabolism through GLS1 up-regulation by sponging miR-214-3p

**DOI:** 10.1007/s00432-024-05736-z

**Published:** 2024-04-25

**Authors:** Jia Liu, Xianbo Zhang, Meijian Yang, Xianghong Zhang

**Affiliations:** 1 Oncology Teaching and Research Office, Hebei Medical University, No. 361, Zhongshan East Road, Shijiazhuang City, 050017 Hebei China; 2 Second Department of Oncology, Hebei General Hospital, No. 348 Heping West Road, Shijiazhuang City, 050051 Hebei Province China; 3 Department of Pathology, The Second Hospital of Hebei Medical University, No. 215, Heping West Road, Shijiazhuang City, 050000 Hebei China

**Keywords:** Colorectal cancer, circCOL1A1, miR-214-3p/GLS1 axis

## Abstract

**Background:**

Circular ribose nucleic acids (circRNAs), an abundant type of noncoding RNAs, are widely expressed in eukaryotic cells and exert a significant impact on the initiation and progression of various disorders, including different types of cancer. However, the specific role of various circRNAs in colorectal cancer (CRC) pathology is still not fully understood.

**Methods:**

The initial step involved the use of quantitative reverse transcription polymerase chain reaction (RT-qPCR) to assess the expression levels of circRNAs and messenger RNA (mRNA) in CRC cell lines and tissues. Subsequently, functional analyses of circCOL1A1 knockdown were conducted in vitro and in vivo through cell counting kit (CCK)-8, colony formation and transwell assays, as well as xenograft mouse model of tumor formation. Molecular expression and interactions were investigated using luciferase reporter assays, Western blot analysis, RNA immunoprecipitation (RIP), and immunohistochemical staining.

**Results:**

The RT-qPCR results revealed elevated levels of circCOL1A1 expressions in CRC tissues and cell lines as compared to the normal counterparts. In addition, circCOL1A1 expression level was found to be correlated with TNM stage, lymph node metastases, distant metastases, and invasion. Knockdown of circCOL1A1 resulted in impaired invasion, migration, and proliferation of CRC cells, and suppressed tumor generation in the animal model. We further demonstrated that circCOL1A1 could act as a sponge for miR-214-3p, suppressing miR-214-3p activity and leading to the upregulation of GLS1 protein to promote glutamine metabolism.

**Conclusion:**

These findings suggest that circCOL1A1 functions as an oncogenic molecule to promote CRC progression via miR-214-3p/GLS1 axis, hinting on the potential of circCOL1A1 as a therapeutic target for CRC.

## Introduction

Colorectal cancer (CRC) has become the second leading cause of cancer-related deaths and the third most prevalent cancer worldwide, resulting in a significant number of annual fatalities (Sung et al. 2020). Despite numerous advancements in diagnosing and treating CRC, patient outcomes are still grim due to late detection, drug resistance, high recurrence rates, and metastasis (Dienstmann et al. 2017). Early detection is crucial for successful intervention, but the current methods have limitations. There is a lack of reliable noninvasive test for CRC early detection, and invasive colonoscopy screening can also generate false positive and negative results (Miller Wilson et al. 2023; Smith et al. 2021). The course of treatment for CRC patients depends on the stage and specific characteristics of the cancer, which include localized treatments (surgery, ablation and embolization, radiation therapy) and systemic treatments (chemotherapy, taregted therapy and immunotherapy) (Adebayo et al. 2023). When considering the limitations of cytotoxic chemotherapy, targeted therapies have been formulated according to the molecular subtyping, such as the agents which target epidermal growth factor receptor (EGFR), vascular endothelial growth factor (VEGF), and DNA mismatch repair pathways (Underwood et al. 2024). Targeted therapy offers several advantages on the systemic CRC treatment, including improved effectiveness and fewer side effects (Sakata and Larson 2022). Nevertheless, an early noninvasive screening strategy based on the reliable molecular biomarkers is pivotal for the early and effective clinical intervention in CRC patients.

CircRNAs (circular RNAs) are emerging as promising candidates for becoming valuable biomarkers in cancer diagnosis and prognosis. These molecules were produced by the back-splicing of certain pre-mRNA transcript, thus possessing covalent-closed structure (Jeck and Sharpless 2014; Rybak-Wolf et al. 2015). CircRNAs are relatively stable and show tissue- and cancer-specific expression. They are also abundant in various body fluids, including blood, serum, and exosomes. These features make circRNAs excellent candidates as biomarkers for pathophysiological conditions (Zhang et al. 2018a). Furthermore, circRNAs are important regulators in different biological processes. In the context of cancer biology, the deregulation of circRNAs have been reported to impinge on different malignant features of cancer cells, such as cell growth, invasion and therapy resistance (Chen et al. 2022; Li et al. 2021). In addition, microRNAs (miRNAs), an evolutionarily conserved group of small noncoding RNAs, can function as downstream mediators of circRNAs (Thomson and Dinger 2016). Previous studies indicated that circRNAs acts as "miRNA sponges" to restrict the activity of miRNA targets, thereby regulating cancer progression (Han et al. 2017; Hsiao et al. 2017; Wei et al. 2017). In CRC, circ100146 was reported to augment the maligant features of tumor cells by repressing miRNA 149 and up-regulating HMGA2 (Liu et al. 2023). CircLDLR was found to be overexpressed in CRC tissues and cells, facilitating the growth and metastasis of CRC cells via miR-30a-3p/SOAT1 axis (Wang et al. 2022). These regulatory modules offer novel perspective on the diagnosis, prognosis and therapy for CRC.

Metabolic reprogramming is a key process sustaining the malignant transformation and metsatsis of tumor cells (Faubert et al. 2020). The recent research has shown that the metabolism of glutamine plays a crucial role in the development of different cancers, such as lung adenocarcinoma, prostate cancer, and B-lymphoma (Faubert et al. 2020; Kery and Papandreou 2020). In this regard, cancer cells seem to become addicted to glutamine catabolism to support hyper-proliferation, invasion, and metastasis abilities (Kery and Papandreou 2020). Glutamine metabolism can be modulated by different circRNAs in cancer cells (Yu et al. 2019). For example, glutaminase (GLS), a key enzyme in glutaminolysis, can be up-regulated by circHMGCS1 in hepatoblastoma cells (Zhen et al. 2019). Similarly, circ0016418 boosts GLS expression in melanoma cells by suppressing the activity of the negative regulator miR-605-5p (Lu et al. 2020a). The understanding of the upstream regulators of glutamine metabolism provides novel target for thwarting the metabolic support in cancer progression.

CircCOL1A1 (hsa_circ_0044556) is a circRNA derived from the transcript of COL1A1 gene (Collagen, Type I, Alpha 1), located on chr17: 48,271,490–48,272,189 (Ma et al. 2021). COL1A1 has been linked to the onset of multiple diseases, including, but not restricted to, breast cancer (Liu et al. 2018), colorectal cancer (CRC) (Zhang et al. 2018b), hepatocellular carcinoma (Ma et al. 2019), and oral squamous cell carcinoma (He et al. 2018). Despite the evidence suggesting the over-expression of circCOL1A1 in gastric cancer (Ma et al. 2021), there is a lack of research investigating its potential involvement in the malignant progression of CRC. In this study, we demonstrated the over-expression of circCOL1A1 and its oncogenic role in CRC. High expression of circCOL1A1 was also associated with a poor prognosis of CRC patient. Silencing circCOL1A1 restricted the function of miR-214-3p, leading to enhanced proliferation, migration, invasion and augmented glutamine metabolism in CRC cells through the upregulation of GLS1 protein levels. Our findings offer significant evidence regarding the regulation of glutamine metabolism by circCOL1A1 in CRC progression.

## Materials and methods

### Clinical specimens

Clinical specimens of CRC tumor samples and para-cancerous normal specimens (*n* = 76 pairs) were obtained from CRC patients undergoing surgery at the Second Hospital of Hebei Medical University between January 2012 and October 2014. The study protocol was approved by the Research Ethics Committee of the Second Hospital of Hebei Medical University. Patients provided informed consent for the collection of their clinical specimens. Notably, all the enrolled patients with CRC had not received radiation or chemotherapy prior to the surgery, and had not been diagnosed with other types of malignancies. Following the surgical resection, the specimens were pathologically verified and promptly stored in liquid nitrogen. The stage categorization of CRC patients were based on the American Joint Committee on Cancer's TNM staging method (7th edition). Table 1 presents a breakdown of clinical and pathological information for the CRC patients based on the expression levels of circCOL1A1 in the tumor specimens. The median expression level of circCOL1A1 in the tumor samples of 76 CRC patients was used as the cut-off to assign the patients into high-expression and low-expression groups.

**Table 1:** Correlation of circCOL1A1 expression with clinicopathologic features of colorectal cancer

Characteristics	Number of cases	circCOL1A1 expression		*P* value
		High (*n* = 38)	Low (*n* = 38)	
Gender				
Male	39	21	18	0.491
Female	37	17	20	
Age (years)				
≤ 60	41	22	19	0.490
> 60	35	16	19	
Tumor size (cm)				0.168
≤ 5	36	15	21	
> 5	40	23	17	
Tumor invasion				
T1–T2	36	11	25	0.001**
T3–T4	40	27	13	
Lymphatic metastasis				
Negative	30	10	20	0.019*
Positive	46	28	18	
Distant metastasis				
M0	66	30	36	0.042*
M1	10	8	2	
Clinical stages				
I–II	32	9	23	0.001**
III–IV	44	29	15	

^*^P < 0.05,**P < 0.01,***P < 0.001

### Cell lines and culture specifications

The human CRC cell lines (HCT116, SW620, SW480, HCT8, and LoVo) and normal colonic epithelial cell line (FHC) were obtained from the Chinese Academy of Science Cell Bank (Shanghai, China) and the FHC human normal colorectal epithelial cell line was sourced from the American Type Culture Collection (ATCC, Manassas, VA, USA). All cell lines were cultured in the appropriate basal medium supplemented with FBS (Gibco, Waltham, MA, USA) and 1% antibiotic/antimycotic solution, then incubated at 37 °C with 5% CO_2_ in a humidified atmosphere. FHC and HCT116 cells were cultured in McCoy's 5A medium (KeyGEN, Nanjing, China), LoVo, and HCT8 cells in DMEM, and SW620 and SW480 cells in L-15 media (KeyGEN, Nanjing, China).

### Lentivirus transduction for stable circCOL1A1 knockdown

Lentivirus particles were produced in 293T cells by transfecting the cells with the pLenti-puro vector carrying circCOL1A1 shRNA or control shRNA (GenePharma, Shanghai, China), together with psPAX2 and pCMV-VSV-G packaging plasmids. The supernatant containing lentiviral particles were then collected after 48 h of transfection. The transduction of HCT116 and SW480 cells was conducted using viral supernatant in the culture medium at the volume ratio of 2:1 in the presence of 8 μg/ml polyberne. 48 h after transduction, cells were selected with 800 ng/ml puromycin for 2 weeks, and the emerging cell clones with successful viral transduction were expanded and stored for further analysis.

### Subcellular fractionation

The Cytoplasmic and Nuclear RNA Purification Kit (Invitrogen, Waltham, MA, USA) was utilized to isolate nuclear and cytoplasmic fractions, and the total RNA in each fraction was purified using Trizol reagent (Beyotime, Beijing, China) based on the supplier's instructions. Subsequently, the presence of circCOL1A1 in the cytoplasmic and nuclear compartment was assessed through RT-qPCR analysis, with glyceraldehyde-3-phosphate dehydrogenase (GAPDH) as the marker for the cytoplasmic fraction and U6 small nuclear RNA (snRNA) for the nuclear compartment.

### Invasion, migration, proliferation, and colony formation assays

The migration and invasion potentials of cells were evaluated via the Transwell inserts (Corning Incorporated, Corning, USA). It is important to mention that, while the protocols for assessing migration and invasion were similar, the key distinction is that the inserts were pre-coated with Matrigel (BD Biosciences, Franklin Lakes, NY, USA) for invasion assays but not necessary for migration. In brief, 2 × 10^5^ cells were seeded into Transwell upper inserts using serum-free media and the lower compartment was filled with media supplemented with 10% FBS. Cells were cultured at 37 °C for 24 h. Subsequently, migrating and invading cells on the insert were fixed and then stained with 0.1% crystal violet (Vicmed, Shenzhen, China) for 15 min. The images were captured at 100× magnification using an Olympus microscope (Olympus, Tokyo, Japan). Finally, ImageJ software was utilized to quantify the number of invading cells. Each experiment was repeated three times.

Cell proliferation was assessed using the Cell Counting Kit (CCK)-8 assay (Sigma-Aldrich, St. Louis, USA). Initially, cells were seeded at a density of 5 × 10^3^ cells/well in a 96-well plate. At indicated time point, 10 μl of CCK-8 solution was added to each well and the plate was further incubated at 37 °C for two hours. The optical density (OD) was measured at 450 nm to quantify cell proliferation capacity. Similarly, the cells were seeded at 2 × 10^3^ cells per well in a 6-well plate and grown in media with 10% FBS for 14 days for the colony formation assay. The medium was changed every four days. The colonies formed were fixed in methanol and stained with 0.1% crystal violet for 15 min. The number of stained colonies was counted to determine the rate of colony formation. All experiment was performed three times independently.

### GLS1 expression vector construction and transfection

The cDNA sequence of human GLS1 gene was synthesized and inserted into the expression vector (pcDNA3.1) by the Shanghai Genomeditech Co. Ltd. (Shanghai, China). Synthetic miRNA mimic, inhibitor and corresponding controls (miR-NC and inhibitor control) were produced by RiboBio (Guangzhou, China). For transfection, cells were firstly seeded in a 6-well plate at a density of 1 × 10^6^ cells/well and incubated until 70% confluence. Cells were then transfected with 200 nm miRNA mimic/inhibitor or 6 μg expression vector using lipofectamine^®^ 2000 (Thermo Fisher Scientific, Inc., Waltham, USA) according to the manufacturer's instructions. Transfected cells were harvested after 72 h for further functional assay and molecular analysis.

### RT-qPCR analysis

As per the manufacturer's guidelines, RNA was isolated from specific cell lines utilizing the Roche Isolation Reagent (Roche, Basel, Switzerland). The generation of complementary DNA (cDNA) was carried out using the Moloney Murine Leukemia Virus reverse transcriptase kit (MMLV RT Kit, ABI, Warrington, UK). RT-qPCR was conducted using the SYBR PCR Kit (Roche) on the ABI 7500 qPCR quantification system. Subsequently, gene expression levels were determined relative to the control gene GAPDH using the delta–delta Ct method. Provided below are the detailed sequences of primers:

circCOL1A1: Forward—CAAGGGTCTGACTGGAAGCC

Reverse – CGAGCTCCTCGCTTTCCTTC

miR-214-3p: Forward—GCGACAGCAGGCACAGACA

Reverse-AGTGCAGGGTCCGAGGTATT

GAPDH: Forward—GGAGCGAGATCCCTCCAAAAT

Reverse—GGCTGTTGTCATACTTCTCATGG

GLS1: Forward—AGTTGCTGGGGGCATTCTTTTAGTT

Reverse—CCTTTGATCACCACCTTCTCTTCGA

### Western blotting

The RIPA lysis buffer (Beyotime, Beijing, China) was used to collect protein samples from cultured cells at 4 °C for 10 min. Subsequently, the mixture was centrifuged at 14,000 g for 10 min at 4 °C. The supernatant was collected for protein measurement by a Bradford assay kit (Zeye Biotech, Shanghai, China). Next, 20 µg of denatured protein sample was loaded into each lane of a 10% sodium dodecyl sulfate–polyacrylamide gel for electrophoresis, followed by the transfer onto a polyvinylidene difluoride (PVDF) membrane. The membrane was then blocked with 5% non-fat milk before incubating overnight at 4 °C with primary antibodies for GLS1 and GADPH (Invitrogen, 1:1000). The next day, the membrane was further probed with horseradish peroxidase (HRP)-linked secondary antibodies (1: 5000, Abcam, Cambridge, UK) for 1 h at room temperature. Protein bands were visualized using BeyoECL PLUS Supersensitive ECL Chemiluminescence Kit (Beyotime, Beijing, China).

### RNA immunoprecipitation (RIP) assay

A Magna RNA-binding protein immunoprecipitation kit (Millipore, Billerica, MA, USA) was used to carry out the RIP experiment, according to the manufacturer’s instructions. Briefly, 10 μg anti-Ago2 or IgG antibodies (Abcam, Cambridge, UK) were immobilized with the protein A/G beads and then incubated with the cell extracts from 1 million cells for 4 h at °C. After washing, the RNA samples on the beads were purified using Trizol reagent and RT-qPCR analysis was conducted to quantify the precipitated RNAs.

### Dual luciferase reporter assay

The predicted interacting sites (WT) or the mutated sequences (MUT) were cloned into the pmirGLO firefly luciferase reporter vector (Promega Corp., Madison, USA). The cells at a logarithmic phase were seeded in the 24-well plates at a density of 1.5 × 10^4^/well. 1 μg WT or MUT reporter vector and 1 μg pRL Renilla control vector were introducted into CRC cells using the Lipofectamine^TM^ 2000 reagent. After 48 h of transfection, a dual-luciferase reporter assay system (Promega) was utilized to detect firefly luciferase activity from the reporter vector and renilla luciferase activity from the control vector, respectively.

### RNA pull-down experiment

The lysates of CRC cells (1 × 10^6^ cells) were collected by IP lysis buffer (Beyotime) and were incubated with 200 nM biotinylated circCOL1A1 probe and control oligos for 2 h at 4 °C. 100 μL M-280 streptavidin magnetic beads (Sigma-Aldrich) was added to the mixture for overnight incubation. A magnetic rod was employed to bring down the magnetic spheres and linked nucleic acids, following which the specimens underwent a total of 4 washes with a high-salt washing solution. The RNA samples from the magnetic pull-down were extracted using Trizol reagent and were subjected to RT-qPCR analysis.

### Immunohistochemical staining

Initially, CRC samples were fixed and encased in paraffin, then cut into 4-μm slices. Following this, the samples were deparaffinized and rehydrated. The activity of peroxidase was blocked by exposing the slides to 95 °C in citrate buffer (0.01 M, pH 6.0) for 20 min. Next, goat serum was applied to the slices for 1 h and the sample was further incubated overnight at 4 °C with anti-Ki-67 antibody (1:500, Abcam, Cambridge, UK). Next day, after the labeling with (HRP)-linked secondary antibody, the sample was treated with 3,3ʹ-diaminobenzidine substrate (DAB; Zhongshan Biotech, Beijing, China) for 5 min for color development. Then, all sections were dehydrated, stained with hematoxylin, and sealed before image capturing under Olympus BX-51 light microscope.

### Detection of glutamine consumption and glutamate

CRC cells with different treatment were grown in the basal medium supplemented with glutamine (2 mM). After 24 h of incubation, the intracellular glutamine and glutamate levels were determined using the corresponding assay kits (Biovision, Milpitas, CA, USA) following the manufacturer's instructions.

### Bioinformatics analysis

The interactions between circCOL1A1 and miRNAs were predicted using the starBase (http://starbase.info/) and circBANK (http://www.circbank.cn/) databases. The potential target miRNAs of GLS mRNA 3's untranslated region (UTR) was identified using the starBase database.

### Tumor xenograft experiments

All animal experiments were approved by the Animal Ethics Committee of Second Hospital of Hebei Medical University. Notably, all animal procedures were performed following the National Institutes of Health's Guide for the Care and Use of Laboratory Animals. Female BALB/c nude mice aged four to six weeks were provided by the HFK Bioscience (Beijing, China). Animals were randomly distributed into the control and circCOL1A1 silencing groups (*n* = 6 animals in each group). To set up the xenograft, 5 × 10^6^ sh-NC HCT116 cells or sh-circCOL1A1 HCT116 cells were injected subcutaneously in the animal along with 200 μl of Matrigel. The formation of xenograft tumor was monitored every week. After 5 weeks, all the mice receiving subcutaneous injections were sacrificed by cervical dislocation. The xenograft tumor samples were harvested for further analysis.

### Statistical analysis

Statistical analysis was conducted using SPSS 17.0 software (SPSS, USA), with GraphPad Prism 7 (La Jolla, USA) being utilized for data visualization. The data were presented as mean and standard deviation (S.D.). Two-tailed Student's *t* test or analysis of variance (ANOVA) was employed to identify significant group differences, with statistical significance set at *P* < 0.05. Association between the expression of two molecules was evaluated using Spearman's correlation analysis. Overall survival (OS) rate was calculated using the Kaplan–Meier method and log-rank test. (*P < 0.05, **P < 0.01, ***P < 0.001, ****P < 0.0001).

## Results

### Elevated circCOL1A1 expression is linked to poor prognosis in CRC patients

**Fig. 1:**
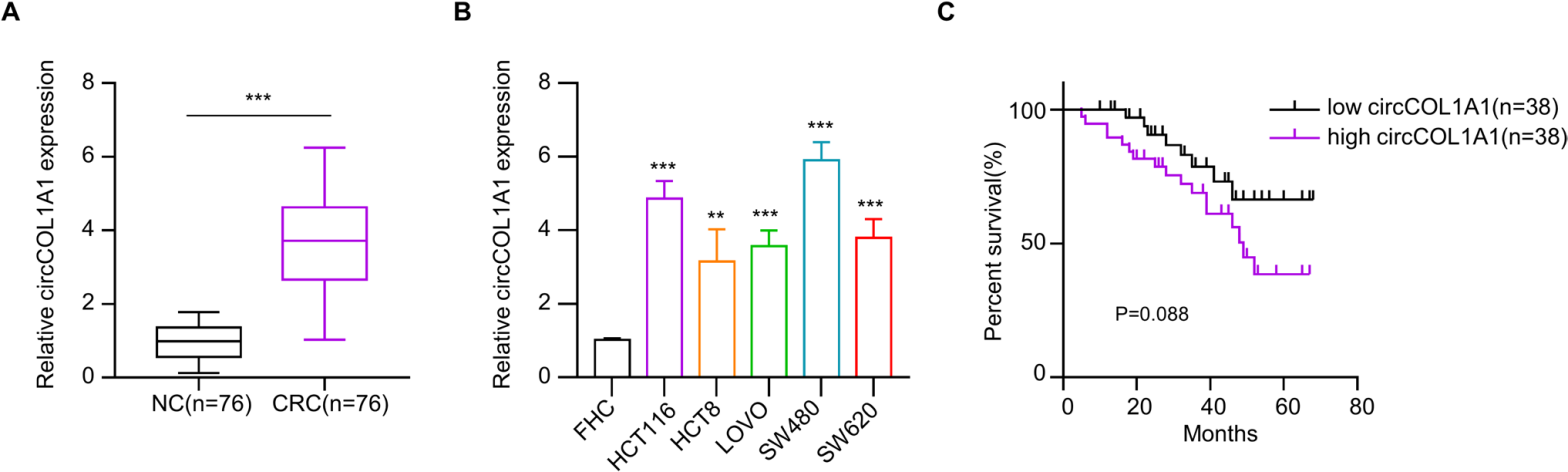
The relative expression level of circCOL1A1 is significantly increased in colorectal cancer (CRC) tissues and cell lines. **A** RT-qPCR analysis shows the circCOL1A1 level in CRC tissues (*n* = 76) and adjacent normal tissues (*n* = 76). **B** RT-qPCR analysis presents circCOL1A1 levels in different CRC cell lines (HCT116, HCT8, LOVO, SW480, and SW620) and normal colonic epithelial cell line (FHC). **C** Overall survival analysis in circCOL1A1 low-and high-expression groups of CRC patients. ** defines *P* < 0.01 and *** presents *P* < 0.001

In order to investigate the pattern of circCOL1A1 expression on CRC, we collected CRC tumor samples and the matched para-cancerous normal tissues from 76 patients. RT-qPCR results revealed significantly elevated circCOL1A1 expression levels in the CRC tumor tissues compared to their corresponding para-cancerous tissues (*P* < 0.001) (Fig. 1A). Likewise, the CRC cell lines exhibited significantly higher circCOL1A1 expression levels compared to the normal colonic epithelial cell line, FHC (*P* < 0.05) (Fig. 1B). The patients were grouped into high- and low-expression category based on the median expression levels of circCOL1A1 in their tumor samples. Survival analysis indicated that patients with heightened circCOL1A1 expression was linked to a worse prognosis (reduced overall survival) in the CRC patients (Fig. 1C). There were no significant associations between tumor size, age, gender, and circCOL1A1 expression levels in CRC patients. However, TNM stage, distant metastasis, and lymphoid metastasis were significantly associated with circCOL1A1 levels (Table 1). Moreover, univariate and multivariate cox analysis also demonstrated that distant metastasis, lymphatic metastasis and circCOL1A1 expression levels are significantly associated with the overall survival of the patients (Table 2).

**Table 2:** Univariate and multivariate Cox analysis of clinical characteristics in relation to overall survival

Variables	Univariate analysis			Multivariate analysis		
	HR	95% Cl	*P* value	HR	95% Cl	*P* value
Gender (male/female)	1.141	0.627–1.943	0.857			
Age (≤ 60/ > 60)	1.026	0.602–1.893	0.529			
Tumor size (≤ 5/ > 5)	1.541	0.892–2.648	0.108			
Tumor invasion (T1–T2/T3–T4)	2.064	0.947–4.013	0.073			
Lymphatic metastasis (Negative/Positive)	1.846	0.711–2.654	0.034*	1.819	0.628–2.628	0.041*
Distant metastasis (M0/M1)	2.908	1.031–4.147	0.016*	2.885	0.958–4.238	0.033*
Clinical stages (I–II/III–IV)	2.542	1.114–3.668	0.041*	2.441	1.217–3.583	0.072
circCOL1A1 expression (High/Low)	4.528	2.152–5.957	0.002*	4.018	2.315–6.157	0.012*

*denotes *p* values smaller than 0.05

### Knockdown of circCOL1A1 suppresses the proliferation, migration, and invasion of CRC cells

**Fig. 2:**
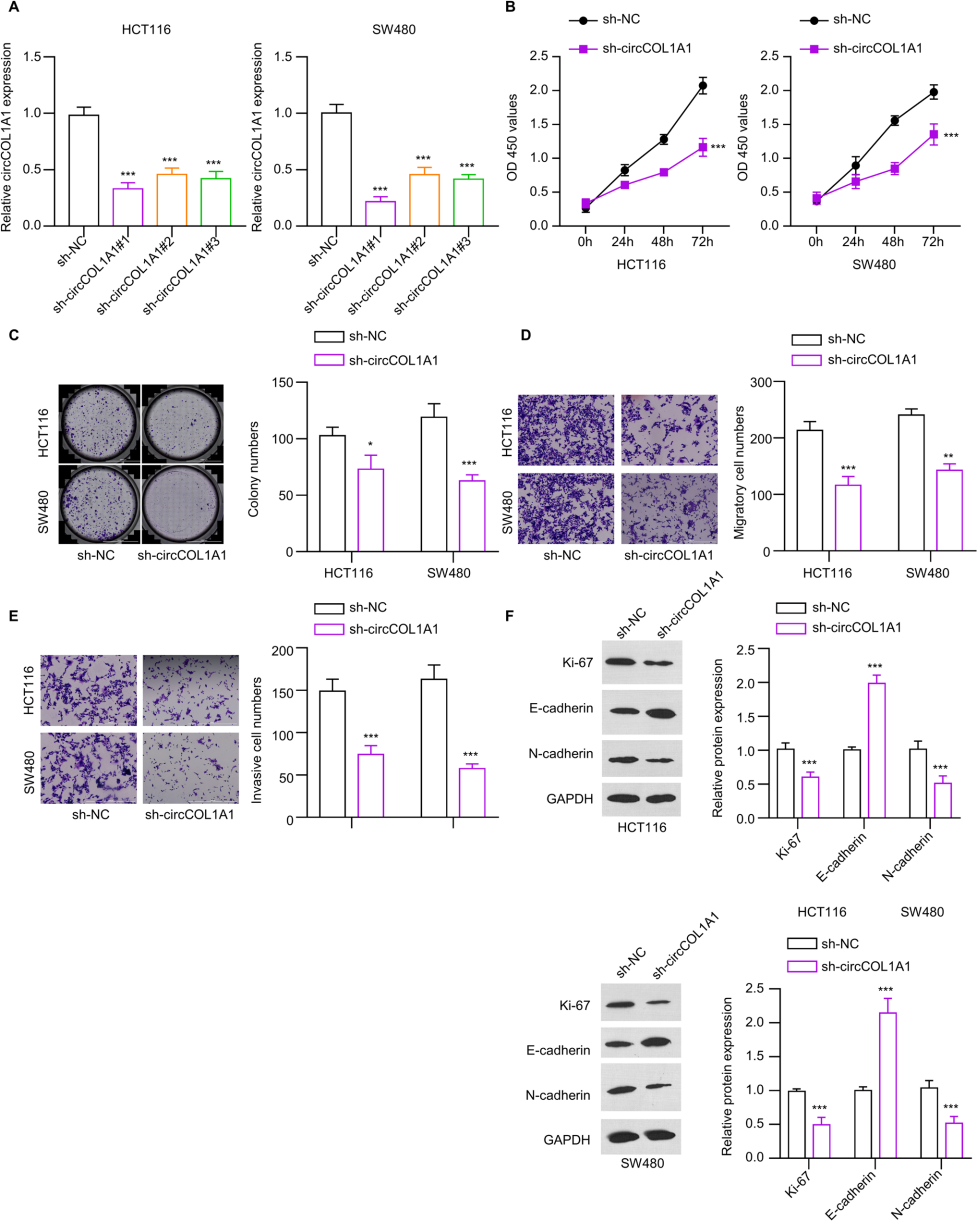
Knockdown of circCOL1A1 suppressed the proliferation, invasion and migration capacities of CRC cells in vitro. **A** RT-qPCR analysis of circCOL1A1 expression in HCT116 and SW480 cells transduced with lentivirus carrying sh-NC or sh-circCOL1A1. **B** CCK-8 assay presents the growth curve of CRC cells in the sh-NC or sh-circCOL1A1 groups. **C** Colony formation, **D** Transwell migration, and **E** invasion assays in CRC cells of the sh-NC or sh-circCOL1A1 groups. **F** Western blot detection of Ki-67, N-cadherin and E-cadherin in CRC cells of the sh-NC or sh-circCOL1A1 groups. ** defines *P* < 0.01 and *** presents *P* < 0.001

The loss-function experiments were conducted to investigate the functional role of circCOL1A1 in CRC cells. Lentiviral transduction were employed to stably silence the circCOL1A1 in two CRC cell lines (HCT116 and SW480) based on shRNA. Following circCOL1A1 knockdown by different shRNAs, RT-qPCR analysis demonstrated decreased expression levels by three circCOL1A1shRNAs. Notably, the knockdown efficiency of sh-circCOL1A1 #1 was the strongest among the three shRNAs (Fig. 2A), which was used for the subsequent loss-of-function experiments. Analysis using the CCK-8 assay showed a decrease in the cell growth after circCOL1A1 knockdown (Fig. 2B). Moreover, there was a notable decrease in clonogenicity in HCT116 and SW480 cells following circCOL1A1 knockdown (Fig. 2C). Similarly, the invasion and migration capacities were hindered after circCOL1A1 knockdown (Fig. 2D and E). Western blot analysis indicated a reduced expression in Ki67 (proliferation marker) and N-cadherin expression (mesenchymal marker) CRC cell lines, while there was an concomitant increase in E-cadherin (epithelial marker) following circCOL1A1 knockdown (Fig. 2F). These data suggest that knockdown of circCOL1A1 suppresses the malignant features of CRC cells, as well as the epithelial-mesenchymal transition (EMT).

### Knockdown circCOL1A1 inhibits glutamine breakdown in CRC cells

**Fig. 3:**
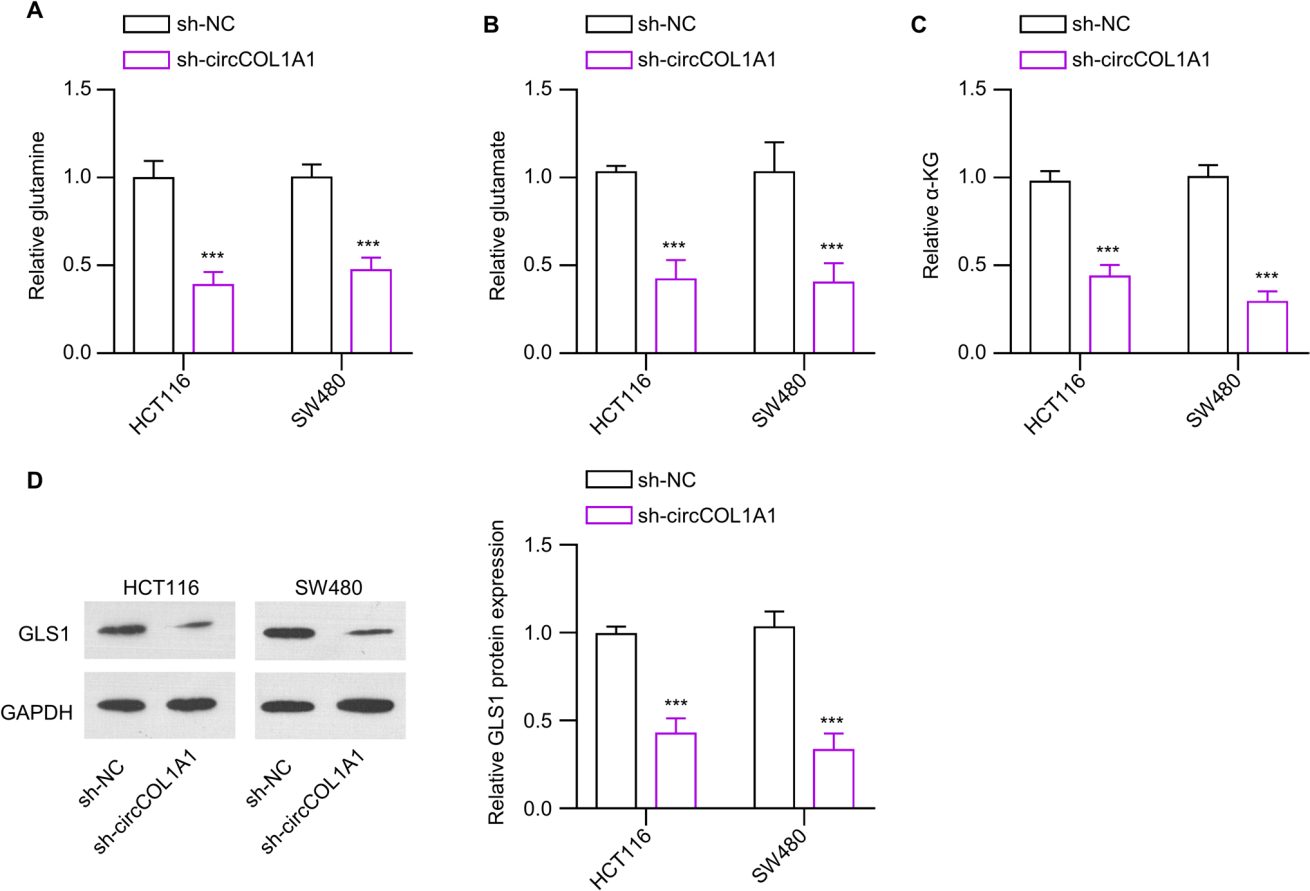
circCOL1A1 knockdown suppresses glutamine metabolism in the CRC cells. **A** Glutamine level, **B** glutamate level, and **C** α-KG production level detection in CRC cells of the sh-NC or sh-circCOL1A1 groups. **D** GLS1 protein expression levels in CRC cells of the sh-NC or sh-circCOL1A1 groups. ** defines *P* < 0.01 and *** presents *P* < 0.001

To investigate the impact of circCOL1A1 on glutamine metabolism (sh-NC and sh-circCOL1A1), the intracellular levels of glutamine, glutamate, and α-ketoglutarate (α-KG) were detected in CRC cells with or without circCOL1A1 knockdown. The findings revealed that the depletion of circCOL1A1 lowered the uptake level of glutamine, impaired the production of glutamate and α-KG synthesis (Fig. 3A–C). Furthermore, the expression of GLS protein (key protein involved in glutaminolysis) was markedly reduced in the sh-circCOL1A1 group of CRC cells (Fig. 3D). Thus, these data suggest that silencing of circCOL1A1 represses glutamine catabolism.

### circCOL1A1 is a negative regulator of miR-214-3p

**Fig. 4:**
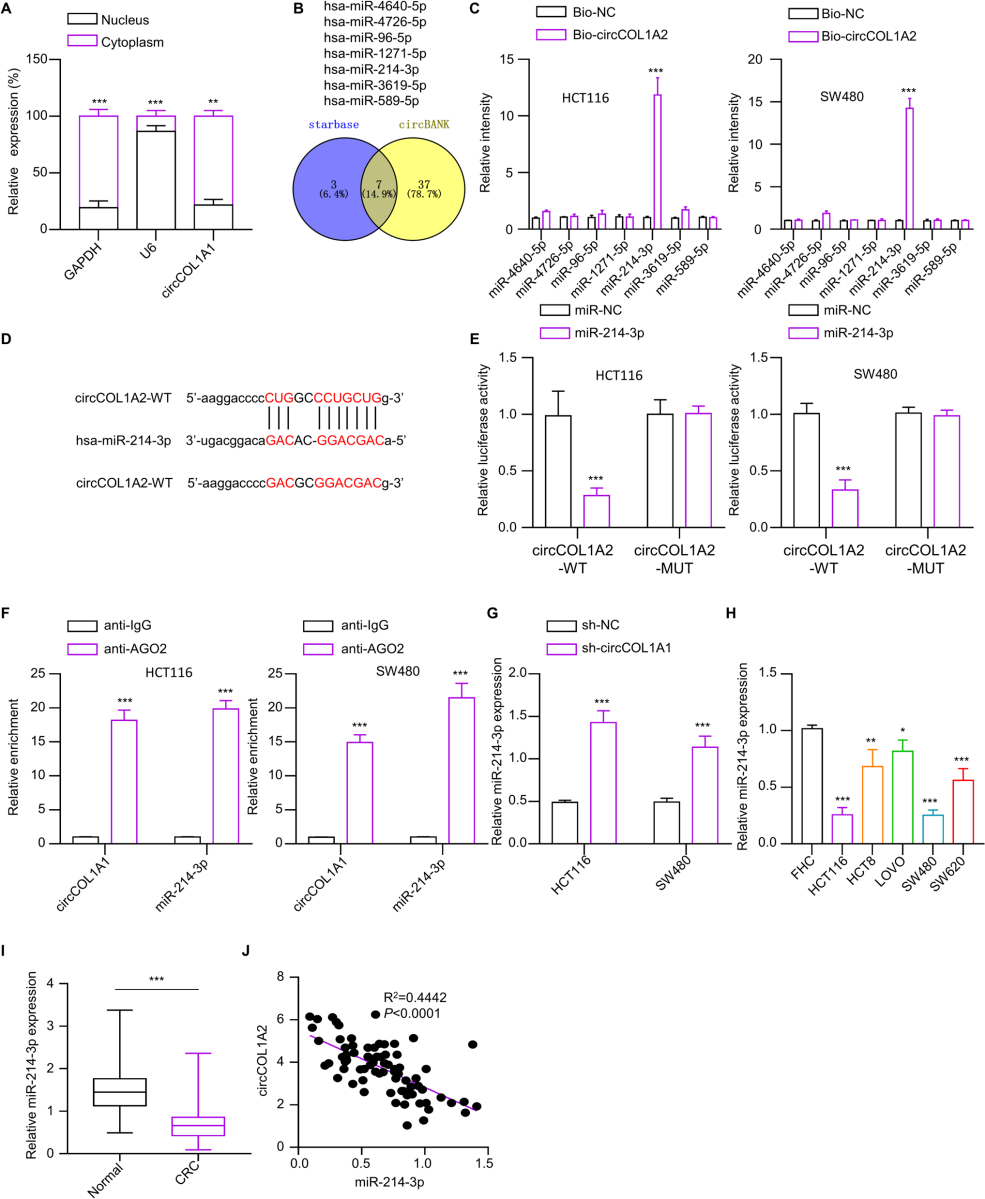
circCOL1A1 sponges miR-214-3p. **A** The subcellular abundance of circCOL1A1 was analyzed by RT-qPCR in HCT116 cells. U6 and GAPDH serve as the nuclear and cytoplasmic controls, respectively. **B** Predicted miRNA targets of circCOL1A1 by starBase and circBank databases. **C** RNA pull-down analysis of miRNA targets using biotin-labeled circCOL1A1 or control probe (NC). **D** The representation shows the putative binding sites (WT) and the mutated sequence (MUT) between circCOL1A1 and miR-214-3p. **E** Dual luciferase reporter assay using WT or MUT luciferase reporters in HCT116 and SW480 cells. **F** Anti-Ago2 RNA-RIP analysis of the physical association between circCOL1A1 and miR-214-3p, with IgG serving as the negative control. **G** RT-qPCR analysis shows the expression of miR-214-3p in CRC cells with or without circCOL1A1 knockdown. **H** RT-qPCR analysis of miR-214-3p levels in different CRC cell lines (HCT116, HCT8, LOVO, SW480, and SW620) and normal colonic epithelial cell line (FHC). **I** RT-qPCt analysis of expression levels of miR-214-3p in CRC tissues (*n* = 76) and adjacent normal tissues (*n* = 76). **J** Pearson’s correlation analysis of circCOL1A1 and miR-214-3p levels in CRC tumor samples. * indicates *P* < 0.05, ** defines *P* < 0.01, and *** presents *P* < 0.001

The subcellular localization of circCOL1A1 was further investigated by examining its expression levels in the nucleus and cytoplasm. The cytoplasm exhibited a predominant concentration of circCOL1A1 expression (Fig. 4A). Seven miRNAs were identified as the potential miRNA targets of from circCOL1A1 public databases, including hsa-miR-4640-5p, hsa-miR-4726-5p, hsa-miR-96-5p, hsa-miR-1271-5p, hsa-miR-214-3p, hsa-miR-3619-5p, and hsa-miR-589-5p (Fig. 4B). In addition, qRT-PCR analysis revealed that biotin-labeled circCOL1A1 could precipitate miR-214-3p in the CRC cell lines HCT 116 and SW 480 (Fig. 4C). The predicted and mutated binding sites between the two molecules is depicted in Fig. 4D. The interaction between miR-214-3p and circCOL1A1 was confirmed through a luciferase reporter assay, where overexpressing miR-214-3p in CRC cell lines resulted in a suppression in luciferase activity of WT reporter (Fig. 4E), indicting their interaction through predicted sequences. RIP-qRT-PCR analysis showed the enrichment of both miR-214-3p and circCOL1A1 with AGO2 antibody in CRC cell lines (Fig. 4F), suggesting their physical association. Knocking down circCOL1A1 led to increased miR-214-3p expression in CRC cells (Fig. 4G). In contrast to circCOL1A1, the expression levels of miR-214-3p were reduced in a range of CRC cell lines, as well as in the CRC tumor samples (Fig. 4H and I). Besides, a negative correlation between miR-214-3p and circCOL1A1 expression levels was found in the CRC tumors (Fig. 4J). The above data indicate that circCOL1A1 is a negative regulator of miR-214-3p in CRC.

### miR-214-3p targets GLS1 expression

**Fig. 5:**
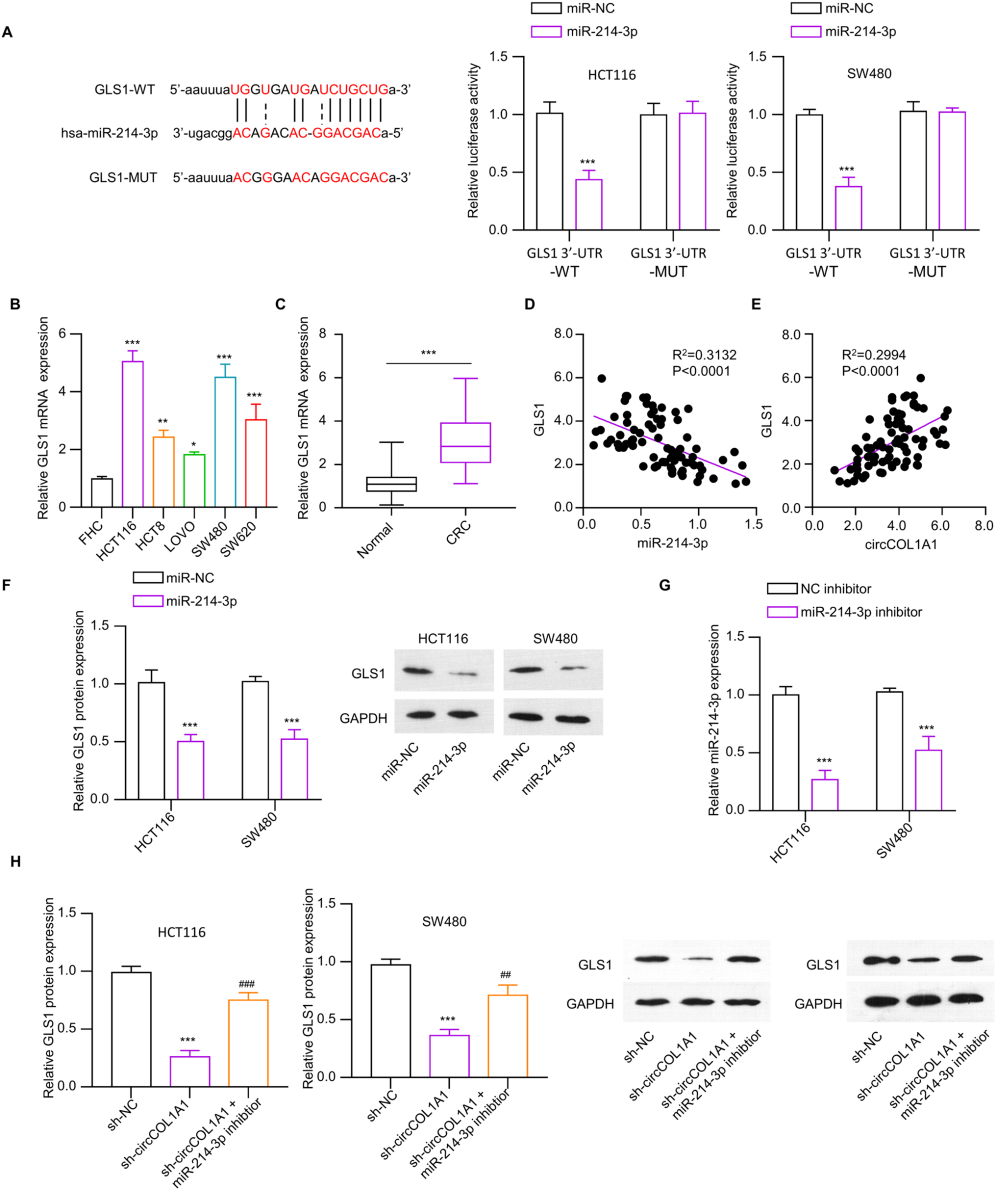
mir-214-3p negatively regulates GLS1. **A** The putative binding sites of miR-214-3p with 3’UTR of GLS1 mRNA, and the dual luciferase reporter validation of their interaction. **B** RT-qPCR analysis of GLS1 expression in different CRC cell lines (HCT116, HCT8, LOVO, SW480, and SW620) and normal colonic epithelial cell line (FHC). **C** RT-qPCR analysis shows the relative expression levels of GLS1 in the CRC tissues (*n* = 76) and adjacent normal tissues (*n* = 76). Correlation analysis of **D** GLS1 and miR-214-3p, and **E** circCOL1A1 and GLS in CRC tumor tissues. **F** Analysis of GLS1 protein levels in CRC cells with miR-214-3p overexpression and miR-NC serves as the control. **G** RT-qPCR analysis of miR-214-3p after the transfection of inhibitor control and miR-214-3p inhibitor. **H** GLS1 protein levels in the CRC cells of sh-NC, sh-circCOL1A1, and sh-circCOL1A1 + miR-214-3p inhibitor groups. * indicates *P* < 0.05, ** defines *P* < 0.01, and *** presents *P* < 0.001

To gain a deeper understanding of the mechanism of action, we conducted a search within the starBase database to identify downstream mRNA targets for miR-214-3p. As illustrated in Fig. 5A, there was putative binding sequences between miR-214-3p to GLS1 mRNA, and their functional interaction was verified by dual luciferase reporter assay. In addition, an assessment of GLS1 expression levels in CRC cell lines and tissues revealed a significantly elevated expression of GLS1 in cancerous cell lines and CRC tissues when compared to the normal counterparts (Fig. 5B and C). Moreover, a positive relationship between GLS1 expression and circCOL1A1, and conversely, a negative correlation between GLS1 expression and circCOL1A1 were identified in CRC tumor samples (Fig. 5D and E). CRC cell lines demonstrated a decrease in GLS1 expression upon overexpression of miR-214-3p (Fig. 5F). In addition, we applied miR-214-3p inhibitor to reduce the expression of miR-214-3p (Fig. 5G). The introduction of miR-214-3p inhibitor could effectively rescue GLS1 expression levels in CRC cells with circCOL1A1 silencing (Fig. 5H). Thus, our data imply that miR-214-3p functions downstream of circCOL1A1 to target GLS1 expression.

### circCOL1A1 regulates the malignant phenotype of CRC cells through miR-214-3p/GLS1 axis

**Fig. 6:**
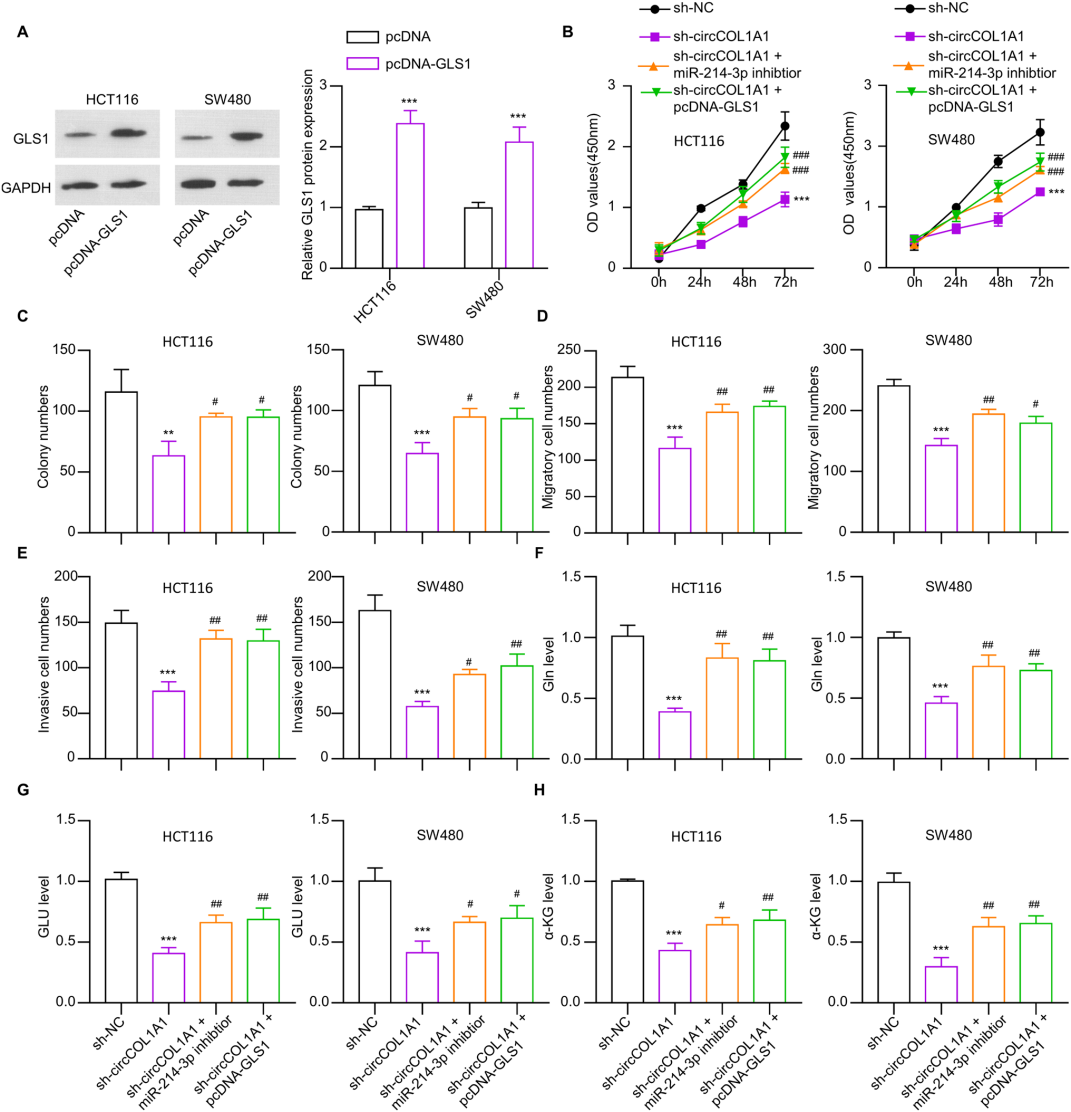
mir-214-3p and GLS1 mediate the function of circCOL1A1 in CRC cells. **A** Western blot analysis presents GLS1 protein levels in CRC cells transfected with pcDNA (control vector) or pcDNA-GLS1 (GLS1 expression vector). Cells were divided into sh-NC, sh-circCOL1A1, and sh-circCOL1A1 + miR-214-3p inhibitor, and sh-circCOL1A1 + pcDNA-GLS1 groups. **B** CCK-8 assay shows growth ability in each group. **C** Colony formation, **D** migration, and **E** invasion assays in each group. **F** Glutamine level, **G** glutamate level, and **H** α-KG production level detection in each group. * indicates *P* < 0.05, ** defines *P* < 0.01, and *** presents *P* < 0.001

Further, rescue studies were conducted to confirm the involvement of miR-214-3p/GLS1 axis in regulating the proliferation, invasion, and glutamine metabolism in the CRC cells. At first, the Western blot analysis confirmed the increased GLS1 level after the transfection of pcDNA-GLS1 expression vector (Fig. 6A). In addition, we showed that transfection of miR-214-3p inhibitor or pcDNA-GLS1 could rescue the growth ability of CRC cells after circCOL1A1 knockdown (Fig. 6B). Similar results were found in the colony formation assay experiment (Fig. 6C). In addition, the introduction of miR-214-3p inhibitor or pcDNA-GLS1 boosted the migratory and invasive capabilities of CRC cells with circCOL1A1 knockdown (Fig. 6D and E). Meanwhile, the transfection of miR-214-3p inhibitor or pcDNA-GLS1 restored the ability of glutamine consumption, glutamate synthesis, and α-KG production in HCT116 and SW480 cells post circCOL1A1 knockdown (Fig. 6F–H). These results suggest that circCOL1A1 regulates the malignant phenotype of CRC cells through mir-214-3p/GLS1 axis.

### circCOL1A1 is required for the tumor formation of CRC cells in vivo

**Fig. 7:**
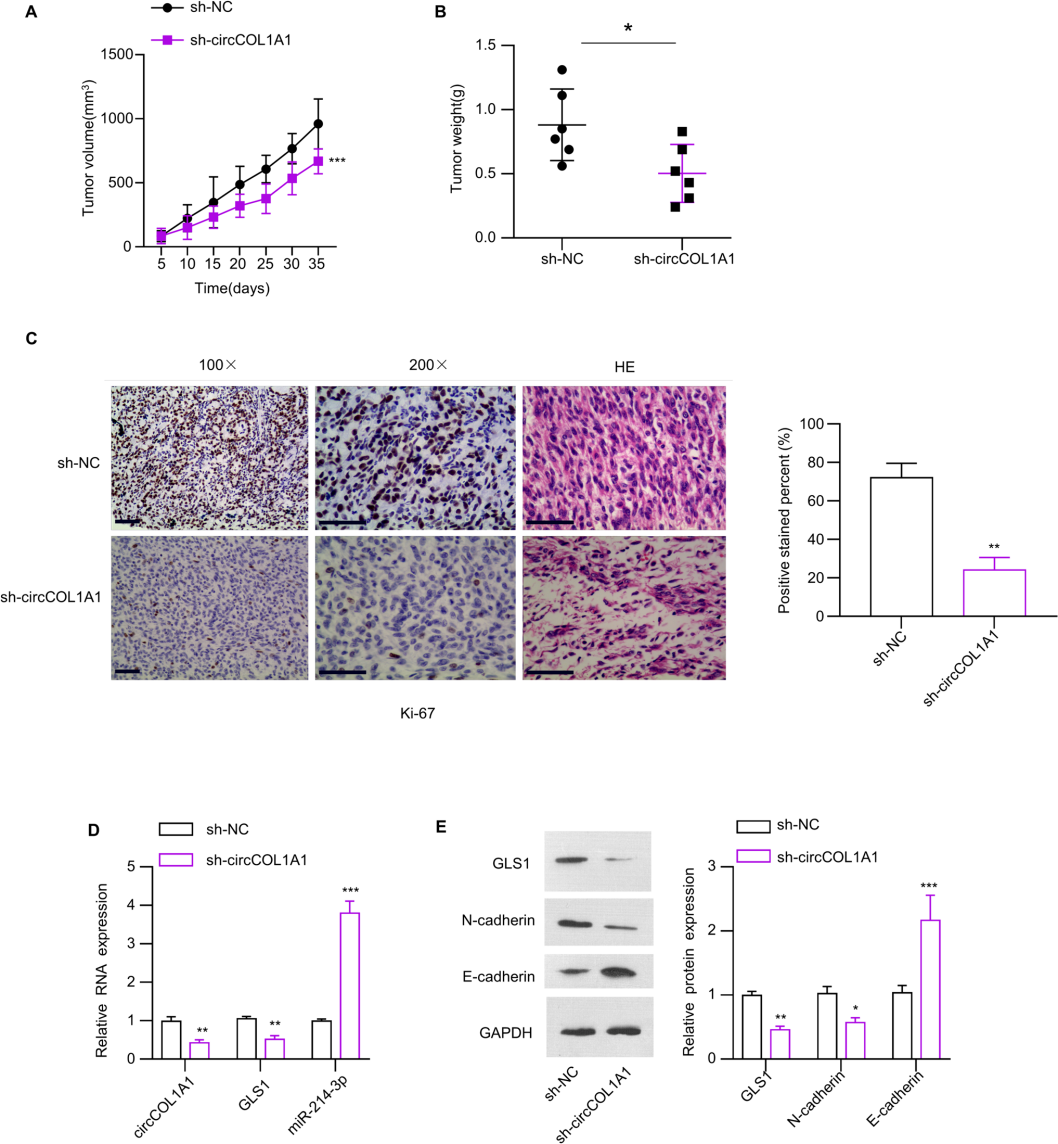
circCOL1A1 is required to support the tumor formation of CRC cells in vivo. Nude mice were subcutaneously injected with HCT116 cells with sh-NC or sh-circCOL1A1. **A** The tumor growth volume curve and **B** the summary of tumor weight. **C** Immunohistochemical staining of Ki67 in the tumor tissues with or without circCOL1A1 knockdown. **D** RT-qPCR analysis of circCOL1A1, miR-214-3p, and GLS1. **E** Western blot analysis of GLS1, E-cadherin and N-cadherin in the tumor samples. * indicates *P* < 0.05, ** defines *P* < 0.01, and *** presents *P* < 0.001

In order to investigate the impact of circCOL1A1 on the tumor generation of CRC cells, we relied on a xenograft tumor model to assess the tumor formation of HCT116 cells carrying sh-NC HCT116 cells or sh-circCOL1A1. Similar to the results from the in vitro analyse, silencing circCOL1A1 suppressed the tumorigenesis in mice (Fig. 7A, B). In addition, Ki-67 expression in tumor tissues. The data revealed that sidelining circCOL1A1 diminished Ki-67 expression in CRC tumor tissues (Fig. 7C). Moreover, a decrease in the levels of circCOL1A1 and GLS1, along with an increase in miR-214-3p expression, was detected in the tumor samples from sh-circCOL1A1 group (Fig. 7D). At protein levels, GLS1 and E-cadherin were reduced and there was an increase in N-cadherin levels in the tumor samples where circCOL1A1 was knocked down (Fig. 7E), suggesting a decrease in tumor aggressiveness.

## Discussion

Recently, several studies have shown that various circRNAs play a role in the progression of CRC by acting as sponges for miRNAs and controlling the expression of genes that encode proteins (Zhou et al. 2021; Yu et al. 2019). In such cases, circRNAs have a significant impact on tumor growth by modulating cellular metabolism, such as glucose metabolism, glutamine breakdown, and lipid metabolism (Yu et al. 2019). Here, we unveiled the mechanism through which circCOL1A1 impinges on glutaminolysis in CRC cells via miR-214-3p/GLS1 axis.

We showed that circCOL1A1 levels were elevated in CRC tumor specimens and cell lines, and circCOL1A1 overexpression seems to contribute to the dismal prognosis in CRC patients. These evidence corroborates the tumor-promoting effect of circCOL1A1 in CRC. Indeed, silencing circCOL1A1 not only undermined the malignant characteristics of CRC cells, but also suppressed the tumor-forming ability in vivo. Of note, our data are consistent with the reported oncogenic role of circCOL1A1 in gastric cancer (Ma et al. 2021). There is also recent evidence showing that exosomal circCOL1A1 derived from CRC facilitate angiogenesis in tumor progression (Hu et al. 2023).

Besides, we demonstrated miR-214-3p as a potential candidate for circCOL1A1 regulation. Its expression was negatively affected by circCOL1A1, and the expression levels were negatively associated with circCOL1A1 in CRC tumors. Inhibiting miR-214-3p rescued the malignancy of CRC cells after circCOL1A1 knockdown. Previous studies indicated that miR-214-3p acts as a tumor suppressor in various tumor types (Li et al. 2016; Fang et al. 2019; Tao et al. 2021). In such a case, miR-214-3p repressed TWIST1 expression in endometrial cancer cells, which suppresses the EMT and metastasis (Fang et al. 2019). miR-214-3p also negatively impacts on tumor growth in lung cancer, and its activity is affected by circCPA4 (Tao et al. 2021). Together, our findings and previous evidence pinpoint miR-214-3p as a key tumor-suppressor in various cancer types (Cagle et al. 2021; Lu et al. 2020b).

Furthermore, we showed that circCOL1A1 silencing impaired cellular ability to uptake glutamine and the subsequent glutamine catabolsim in CRC cells. GLS1, a rate-limiting enzyme in glutaminolysis was found to be negatively regulated by miR-214-3p and positively regulated by circCOL1A1. Forced GLS1 expression could rescue glutamine metabolism and the agressive features of the CRC cell lines. In line with our findings, several lines of previous evidence revealed the overexpression of GLS1 in the CRC tissues (Huang et al. 2014; Xiang et al. 2019; Xu et al. 2015).

Generally, metabolic processs not only supports cellular function, but also affect gene expression regulation (Lu and Thompson 2012). Glutaminolysis generates various metabolites like glutamate, α-KG, 2-hydroxyglutarate, succinyl-CoA, fumaric acid, acetyl-CoA, citric acid, HMG-CoA, and pyruvic acid, serving as precursors in nucleotide, amino acid, and lipid synthesis and metabolites for epigenetic modifications like methylation and acetylation (Chisolm and Weinmann 2018). GLS, a crucial enzyme in glutamine metabolism, acts as a tumor promoter in multiple cancers (Masisi et al. 2020; Matés et al. 2013). When GLS is silenced in prostate cancer cells, cell growth is inhibited and apoptosis induced as a result of Wnt/β-catenin pathway inactivation (Zhang, et al. 2019). GLS dysregulation also hinders glioma cell growth by triggering oxidative stress (Martín-Rufián et al. 2014). Our study showed that GLS1 was up-regulated in CRC tumors, and forced GLS1 expression counteracted the inhibitory effects of circCOL1A1 knockdown on CRC. Thus, targeting circCOL1A1 is anticipated to become an approach to modulate glutamine metabolism and suppress the malignant progression in CRC.

## Conclusion

To summarize, our research revealed that circCOL1A1 contributes to CRC progression by promoting glutamine metabolism. circCOL1A1 overexpression suppresses miR-214-3p to enhance GLS1 level. Silencing circCOL1A1 impeded the aggressiveness of CRC cells and the tumor growth both in animal model. These findings imply that targeting the circCOL1A1/miR-214-3p/GLS1 axis could offer innovative strategies for treating CRC.

## Data Availability

The datasets used and/or analyzed during the current study are available from the corresponding author via email request.
